# The forgotten duo: narrow-band imaging plus Lugol iodine chromoendoscopy in the early detection of esophageal squamous cell carcinoma

**DOI:** 10.1016/j.igie.2025.11.008

**Published:** 2025-12-09

**Authors:** Diego Cadena-Aguirre, Mimi Tan, Igor Valdeir Gomes de Sousa, Evandro Sobroza de Mello, Sérgio Barbosa Marques, Fauze Maluf-Filho

**Affiliations:** 1 Gastrointestinal Endoscopy Unit, Hospital das Clínicas, University of São Paulo School of Medicine, São Paulo, Brazil; 2 Section of Gastroenterology and Hepatology, Department of Medicine, Baylor College of Medicine, Houston, Texas, USA; 3 Department of Pathology, Instituto do Câncer do Estado de São Paulo, University of São Paulo, São Paulo, Brazil

A 75-year-old man with long-standing untreated idiopathic achalasia presented with progressive dysphagia, regurgitation, and weight loss. His Eckardt score was 8. He was referred for upper endoscopy and evaluation for peroral endoscopic myotomy.

White-light endoscopy (WLE) showed a subtle mucosal irregularity in the mid esophagus (Fig. 1). After spraying 2.5% Lugol iodine solution (diluted from 5% at a 1:1 ratio with normal saline), a 4-cm Lugol-voiding lesion (LVL) involving approximately 40% of the esophageal circumference was revealed. A faint pink color sign was identified within the LVL under WLE, suggesting early neoplasia (Fig. 2). Then, narrow-band imaging (NBI) revealed a distinct 7-mm zone within the LVL that exhibited the “metallic silver sign”—a sharply demarcated, silvery-metallic color change (Fig. 3, Video 1, available online at www.igiejournal.org). Targeted biopsy specimens of this zone confirmed high-grade squamous intraepithelial neoplasia (Fig. 4), whereas biopsy specimens from the rest of the LVL without the silver sign showed only low-grade dysplasia.

**Figure 1:**
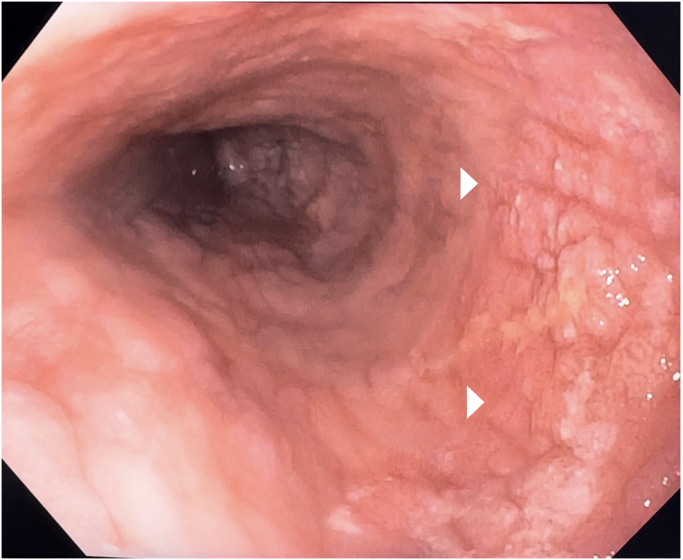
White-light endoscopy showing suspicious area (*arrowheads*).

**Figure 2:**
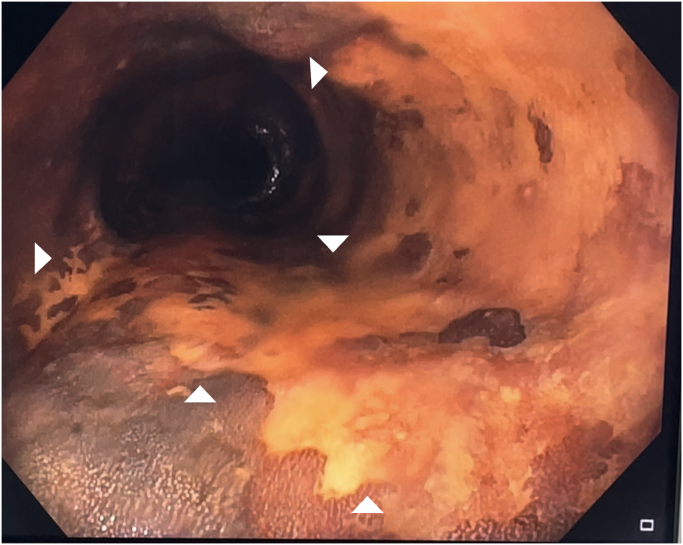
Lugol-voiding lesion (*arrowheads*).

**Figure 3:**
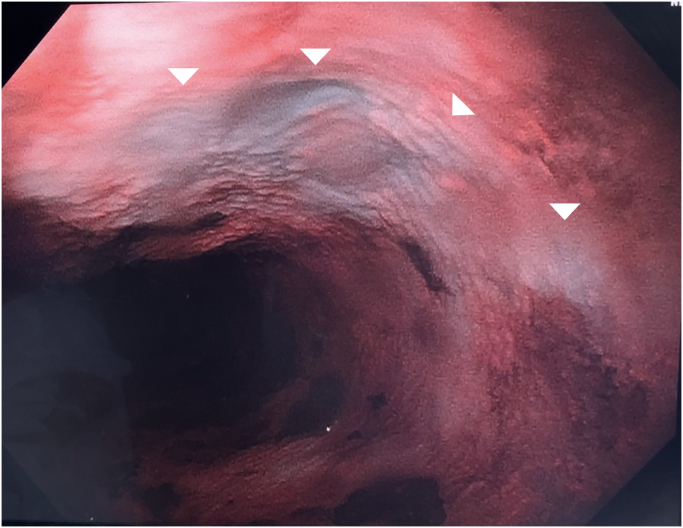
Metallic silver sign (*arrowheads*).

**Figure 4:**
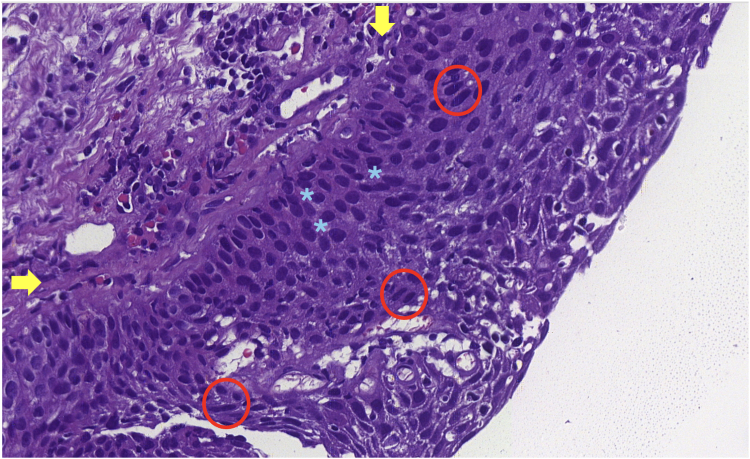
High-power view (hematoxylin and eosin, original magnification ×40). Mitotic figures are observed (*asterisks*). The epithelial nuclei are enlarged, pleomorphic, and hyperchromatic (*circles*). Dysplastic changes involve the entire epithelial thickness above the basement membrane, without invasion into the submucosa (*arrows*).

This case illustrates how combining NBI with Lugol chromoendoscopy enhances the detection of esophageal squamous neoplasia. WLE alone has limited sensitivity, around 50% for early squamous neoplasia.^1^ Lugol chromoendoscopy improves sensitivity to 100%, but its specificity is around 81.2% per patient and 37% per lesion, because of false positives from inflammation or other benign changes.^2^, ^3^, ^4^ NBI alone offers sensitivity between 88% and 94% and specificity of 88% per patient and 65% per lesion.^4^

Interestingly, the metallic silver sign described when using NBI after Lugol staining has been proposed as a visual marker of cancerous lesions, with 1 study reporting a diagnostic accuracy of 98.4% for squamous cell carcinoma.^5^ Although the pink color sign indicates areas suspicious for neoplasia through a subtle pinkish discoloration, the metallic silver sign appears as a clearer, silvery-metallic hue under NBI, offering superior contrast and easier recognition.^5^ This specific finding may help overcome the limitations of Lugol low specificity by focusing biopsies on the most suspicious areas within large voiding zones.

Although virtual chromoendoscopy techniques such as NBI may not be universally available, their use in combination with conventional Lugol chromoendoscopy can be particularly valuable when accessible, because this approach maximizes diagnostic yield. The combined use of NBI and Lugol chromoendoscopy should be revisited, particularly in high-risk patients and in resource-limited settings, where early diagnosis of esophageal squamous cell carcinoma remains a challenge.

## Patient consent

Written informed consent was obtained from the patient for the publication of this case and all accompanying images.

## Disclosure

The following author disclosed financial relationships: F. Maluf-Filho has served as a consultant for Olympus, Boston Scientific, Microtech, and Cook. All other authors disclosed no financial relationships.

## Appendix

Video 1 The metallic silver sign: narrow-band imaging after Lugol chromoendoscopy for early esophageal squamous neoplasia.
